# A Modified Aging Kinetics Model for Aging Condition Prediction of Transformer Polymer Insulation by Employing the Frequency Domain Spectroscopy

**DOI:** 10.3390/polym11122082

**Published:** 2019-12-12

**Authors:** Jiefeng Liu, Xianhao Fan, Yiyi Zhang, Hanbo Zheng, Zixiao Wang, Xixi Zhao

**Affiliations:** College of Electrical Engineering, Guangxi University, Nanning 530004, Guangxi, China; liujiefeng9999@163.com (J.L.); xianhao_fan@163.com (X.F.); hanbozheng@163.com (H.Z.); zixiaowang1996@163.com (Z.W.); blairxixi@163.com (X.Z.)

**Keywords:** transformer polymer insulation, Frequency Domain Spectroscopy (FDS), aging kinetics model, aging condition prediction, degree of polymerization (*DP*)

## Abstract

The aging kinetics model is of great interest to scholars since it is capable of describing the variation law between the degree of polymerization (*DP*) and the aging duration of transformer polymer (cellulose) insulation. However, it is difficult to determine the moisture content inside the transformer polymer insulation without destroying it, so that the model parameters cannot be confirmed. Such limitation greatly restricts its application. It is interesting to note that as long as the moisture content of the transformer polymer insulation could be characterized (replaced) by a certain feature parameter, the above issue will be solved naturally. The existing researches indicate that the Frequency Domain Spectroscopy (FDS) is sensitive to moisture. Consequently, the feature parameter that could characterize the moisture inside transformer polymer insulation (extracted from the FDS curve) can be used to report a modified aging kinetics model, which could perform the aging condition prediction of transformer polymer insulation under various test conditions, including aging duration, aging temperature, and initial moisture. In that respect, the average relative error of prediction results of prepared samples is equal to 7.41%, which reveals that the reported model might be serviced as a potential tool for the aging condition prediction of transformer polymer insulation.

## 1. Introduction

The aging condition of transformer polymer insulation determines the overall service life of the transformer. Existing researches show that the degree of polymerization (*DP*) can be serviced as the most direct and effective indicator for reflecting the aging condition (mechanical strength) of polymer insulation [1,2,3]. Reviewing the published researches, we found out that the method for predicting (or measuring) the *DP* value of transformer polymer insulation consists of several prevailing approaches, such as the direct method (viscosity testing) [4] and indirect methods (including the Frequency Dielectric Spectroscopy (FDS) [5,6,7,8,9] technique, aging kinetics model [10,11,12], etc.). Although the viscosity test method is accurate enough, it is not appropriate for analyzing the field equipment due to its destructiveness. Consequently, the indirect method is of great interest to scholars in contrast with the direct method.

It is widely accepted that the aging by-products, such as moisture, acids, alcohols, aldehydes, etc., produced in the degradation process of transformer polymer insulation could regularly affect its FDS [13]. Therefore, the qualitative or quantitative analysis of the *DP* value of polymer materials can be realized by the relevant feature parameter extracted from the FDS data. In literature [14,15], the integral value of the FDS curve is computed and applied as the feature parameter for achieving the above goal. In addition, the equivalent circuit model that is capable of analyzing the corresponding aging condition is also reported, such as the Cole–Cole model [16], the Havriliak–Negami model [17], the Davidson–Cole model [18], and the Dissado–Hill model [19]. However, it is a fact that the contribution of the aging condition on measured FDS data could be easily covered by the moisture effect [13,14,15]. Therefore, it is rather difficult to accurately analyze the aging condition of the transformer polymer insulation and merely rely on the FDS technique in case the inside moisture is too high.

The published researches pointed out that the aging kinetics model can be employed to describe the degradation process of transformer polymer insulation under different conditions, including temperature, aging duration, and especially the initial moisture content *mc%* [20,21,22,23,24,25]. Thus, the discussion of the aging kinetics model can make up for the limitation of the FDS technique. At present, the research on the aging kinetics model of polymer insulation is mainly based on the Ekamstam equation [24]. Furthermore, Emsley et al. found that the degradation rate of polymer (cellulose) materials will not always have a constant attributed to the leveling-off degree of the polymerization (LODP) phenomenon [21,22,23]. A second-order kinetics model is thus proposed [23,25]. The findings reveal that the second-order kinetics model can accurately describe the variation law of the *DP* value of polymer materials under different reaction conditions. Therefore, as long as the model parameters are determined, the above model can be applied to accurately predict the *DP* value of the polymer insulation materials. However, it is difficult to determine the moisture content inside the unknown sample without destroying it for sampling, which makes the model parameters also uncertain. Such limitation greatly restricts its application on aging condition prediction.

In view of these issues, this research attempts to report a modified aging kinetics model for aging condition prediction by employing the FDS technique, which aims to overcome the mentioned limitations. In the present research, depending on the combination of the advantages of both FDS (sensitive to moisture content) and the aging kinetics model (capable of describing the variation law of *DP*), an available modified model is proposed. Findings indicated that the proposed model could be utilized to predict the aging condition of transformer polymer insulation under various test conditions, including diverse aging temperature, aging duration, and initial moisture content. Moreover, the average relative error of prediction results of oil-immersed polymer (cellulose) pressboards is less than 10%, which further verify the accuracy and feasibility of the reported model. In that respect, the present contribution reveals that the reported modified aging kinetics model might be serviced as a potential and effective tool for aging condition prediction.

## 2. Sample Construction

The transformer polymer (cellulose) insulation material (pressboard) and mineral oil are utilized in this paper so as to investigate its frequency response characteristic under different insulation conditions. Therefore, a series of experiments were carried out under controlled laboratory conditions, and the oil-immersed polymer (cellulose) pressboards with various insulation conditions were later prepared. The details of both polymer pressboard and oil are described in Table 1.

**Figure 1:**
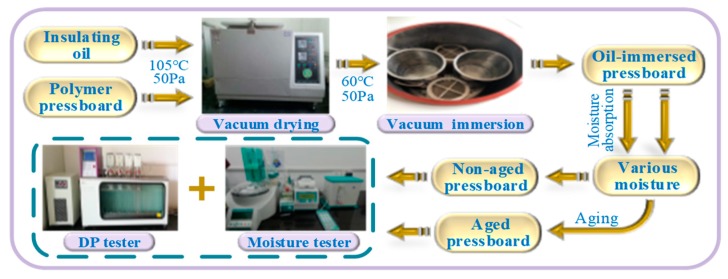
The experimental schedule of oil-immersed polymer (cellulose) pressboard.

The pressboards with various initial moisture contents were completed by moisture absorption. Then, the moisture balance distribution process was later performed at 45 °C and lasted for 48 h so as to perform the FDS test. Subsequently, the *DP* value and moisture content obtained by *DP* tester (by means of viscosity tester, Sierda scientific instrument co., LTD, Shanghai, China) and moister tester (by means of Karl Fischer Titration, Wantong co., LTD, Switzerland) can be found in Figure 1.

## 3. The Establishment of the Traditional Aging Kinetics Model of Transformer Polymer Insulation.

### 3.1. The Derivation of the Traditional Aging Kinetics Model

Cellulose material is a kind of natural organic polymer homopolymer. Its basic structural unit is glucose monomer (C_6_H_10_O_5_), and its repeating unit is cellobiose, its general formula is (C_6_H_10_O_5_)*_n_* in which *n* is known as the degree of polymerization (*DP*) [11,12]. The adjacent glucose monomers are linked by 1-4-β glycoside bond, the degradation process of cellulose materials is actually completed by the break of the glycoside bond. In the course of the operation, the glycoside bond of the cellulose materials gradually breaks due to the suffered stresses, including thermal, oxidation, and hydrolysis stress. In this case, the average number of glucose monomers in each cellulose fiber decreased. Consequently, the corresponding *DP* value decreased with the increase of aging duration.

The kinetics model is widely used to describe the change law of *DP* of polymer materials under the thermal stress [20,21,22,23,24,25]. If *N* is the total number of glucose monomers in the polymer insulation, and *M*_t_ is the total number of polymer molecules when the aging duration reaches time *t*, then the corresponding average value of *DP* of polymer insulation can be regarded as *DP*_t_.

(1) $$DP_{t}=\frac{N}{M_{t}}$$

Chain scission number (CSN) is defined as the times of broken chains per polymer chain during the aging of polymer insulation, the formula for calculating the CSN is as follows:

(2) $$CSN=\frac{DP_{0}}{DP_{t}}-1$$

where *DP*_0_ is defined as the initial *DP* value, which is assumed to be 1100 in this paper. The scission fraction of the polymer unit (SFCU) is defined as the ratio of glucose monomer broken in a polymer chain to the total number of glucose monomers.

(3) $$SFCU=\frac{CSN}{DP_{0}}=\frac{1}{DP_{t}}-\frac{1}{DP_{0}}$$

The existing research on the kinetics model of polymer degradation is mainly based on the aging kinetics model of linear high molecular polymers. These researches suggest that the degradation of polymer belongs to a random chain scission reaction (the probability of breaking each bond between polymer fibers is equal) [20,24]. Further, the degradation rate *k* is considered to be a constant over a certain temperature range. Thus, a kinetics equation for describing the variation law between the *DP* value and the aging duration of polymer insulation materials is proposed [24].

(4) $$\frac{1}{DP_{t}}-\frac{1}{DP_{0}}=k\cdot t$$

However, Heywood [26] pointed out that as long as the *DP* value of polymer (cellulose) insulation materials drops to a certain value, its trajectory will gradually deviate from the first-order kinetics model. In this case, the chemical reaction rate is no longer a constant, which is also known as the leveling-off degree of polymerization [23]. In other words, the parameter *k* shown in Equation (4) is a variable that changes with the alteration of aging time, which can be represented by Equation (5).

(5) $$k(t)=\underset{\Delta t\to 0}{lim}\frac{m_{t}}{N\cdot \Delta t}=k_{1}\cdot e^{-k_{2}\cdot t}$$

From Equation (5), parameters *k*_1_ and *k*_2_ are equal to a constant, which are both related to the initial moisture content inside the polymer insulation materials. If there exists a total of *m* glycosidic bond breaks in the polymer molecule per unit time, the average degree of polymerization *DP_t+_*_Δ*t*_ at *t*+Δ*t* can be written as the following form:

(6) $$DP_{t+\Delta t}=\frac{N}{M_{t}+m}$$

Moreover, the change amount in the *DP* value (△*DP_t+_*_△_*_t_*) is shown in Equation (7).

(7) $$\begin{array}{l} \Delta DP_{t+\Delta t}=\frac{N}{M_{t}+m}-\frac{N}{M_{t}}\\ =-\frac{N\cdot m\cdot \Delta t}{(M_{t}+m)\cdot M_{t}\cdot \Delta t} \end{array}$$

The simultaneity of Equations (1) and (7) provide an expression characterizing the rate of decrease in the *DP* value of polymer insulation materials.

(8) $$\begin{array}{l} DP_{t}^{\prime }=\underset{\Delta t\to 0}{lim}\frac{\Delta DP_{t+\Delta t}}{\Delta t}=\underset{\Delta t\to 0}{lim}-\frac{N^{2}m}{(M_{t}+m)\cdot M_{t}\cdot N\cdot \Delta t}\\ \begin{array}{ccc} & & \approx \underset{\Delta t\to 0}{lim}-\frac{N^{2}m}{M_{t}{}^{2}\cdot N\cdot \Delta t} \end{array}=-DP_{t}^{2}\cdot \underset{\Delta t\to 0}{lim}\frac{m}{N\cdot \Delta t}=\frac{dDP_{t}}{dt} \end{array}$$

Substituting Equation (5) into Equation (8), the Equation (8) can be expressed by another form, shown in Equation (9).

(9) $$\frac{1}{DP_{t}^{2}}dDP_{t}^{}=-k(t)dt=-k_{1}\cdot e^{-k_{2}\cdot t}dt$$

From Equation (9), the formula for expressing the relationship among the *DP*, reaction rate, and reaction duration is obtained by employing the integral operation.

(10) $$\begin{array}{l} \int_{DP_{0}}^{DP_{t}}\frac{1}{DP_{}^{2}}dDP=-k_{1}\cdot e^{-k_{2}\cdot t}dt\\ \Rightarrow \frac{1}{DP_{t}}-\frac{1}{DP_{0}}=\frac{k_{1}}{k_{2}}(1-e^{-k_{2}\cdot t}) \end{array}$$

The above formula is known as the second-order aging kinetics model, which is utilized for describing the quantitative relationship between the *DP* value and the reaction time of polymer materials. The second-order kinetics model can accurately reflect the change of the polymerization degree of polymer insulation overall aging processes, and thus has been widely reported [21,23]. However, since the aging rate *k*(*t*) is an attenuation function used to describe the slowing down of the degradation rate of polymer materials when involving the leveling-off degree of polymerization (LODP) phenomenon, it inevitably lacks a clear physical meaning [25]. Afterwards, depending on the analysis of polymer degradation at the molecular level, a kinetics model based on the “percentage *DP* loss” of polymer materials [25].

(11) $$\omega (t)=1-\frac{DP_{t}}{DP_{0}}=\omega_{DP}\cdot (1-e^{-k_{DP}\cdot t})$$

From Equation (11), *ω_DP_* is assumed as the amplitude of the attenuation function, parameter *k_DP_* is named as the degradation rate of polymer, *ω*(*t*) represents the capacity of the “*DP* loss”. Thus, different aging conditions can be represented by the diverse values of *ω*(*t*). If the polymer material has not been degraded so far, the corresponding *ω*(*t*) is equal to 0, and the value is equal to 1 in the case that polymer materials are completely degraded.

### 3.2. The Aging Kinetics Model for Aging Condition Analysis

In this part, several oil-immersed polymer pressboards (shown in Table 1) with different initial moisture content (including 0.78%, 1.32%, 2.07%, 2.74%, and 3.82%) are firstly prepared. Then, the above pressboards are utilized to perform the alternate thermal aging experiment at 150 °C. The *DP* values of the same pressboard with the different aging duration are recorded in the course of the aging experiment. If the horizontal axis and vertical axis are represented by aging duration (hours) and *DP* values, respectively, the resulting picture that adopted to describing the variation law between aging duration and *DP* values can be easily plotted. 

**Figure 2:**
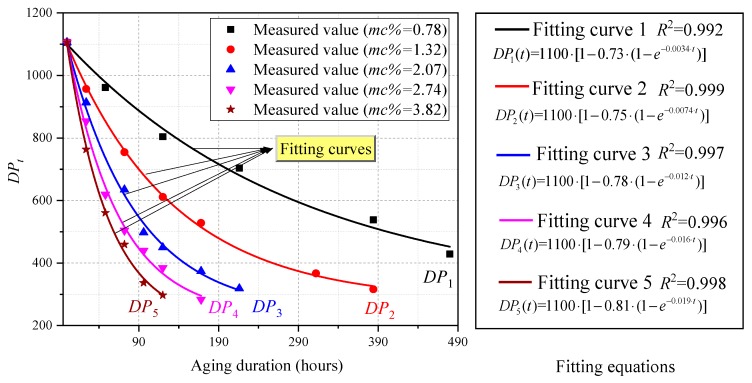
The variation law of the degree of polymerization (*DP*) value vs. aging duration.

Moreover, it is clear that Equation (11) can be utilized to describe the degradation process of polymer insulation. Provided that Equation (11) is adopted to fitting the presented variation law of *DP* vs. aging duration, the parameters (*ω_DP_* and *k_DP_*) in the formula can be naturally computed, and results are shown in Figure 2.

It is obvious that the higher the initial moisture content inside the polymer insulation, the faster the *DP* value decreases, and the less time it takes for the curve to become flat. Meanwhile, the impact generated by the initial moisture content can be also observed in the values of computed parameters. These values and error bursts (*EB*) are shown in Table 2, and the significant figures in the experiment are reserved for 2 digits.

**Figure 3:**
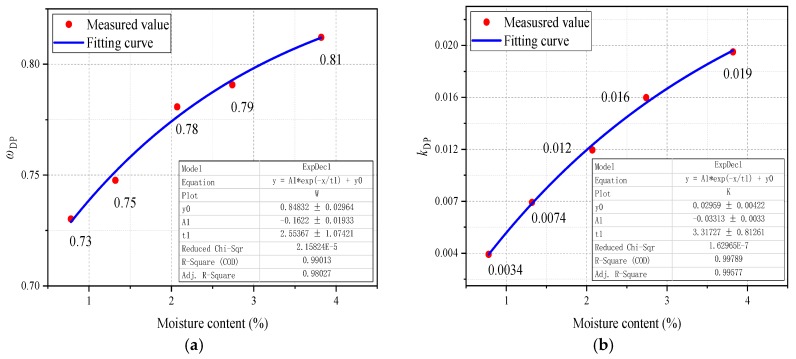
The fitting curves of the parameters in the kinetics model. (**a**) *ω_DP_*, (**b**) *k_DP_*.

The values of parameters shown in Table 2 are subjected to present an increasing trend with the rising initial moisture content. Such the phenomenon has also pointed out that the quantitative relationship of *mc%* vs. *ω_DP_* and *mc%* vs. *k_DP_* can be studied by fitting analysis. The fitting curves and fitting equation can be found in Figure 3 and Table 3, respectively, where the *RSS* and *R*^2^ represent the residual sum of squares and determination coefficient. The closer the value of *RSS* is to 0 and the closer the value of *R*^2^ is to 1, the better the fitting results.

It is obvious that equations shown in Table 3 could describe the functional relationship between relevant parameters (*ω_DP_* and *k_DP_*) and initial moisture content. It means that the higher the moisture content, the larger the value of the *ω_DP_* and *k_DP_*, and thus the faster the degradation rate of polymer insulation materials. By employing a combination of the formulas shown in Equation (11) and Table 3, a universal model for estimating the remaining life *L*(*t*) [27,28] of transformer polymer insulation under any initial moisture content is readily obtained, which can be expressed in the form of Equation (12).

(12) $$L(t)=-\frac{Ln(1-\frac{1-DP_{t}/DP}{\omega_{DP}(mc\%)})}{k_{DP}(mc\%)}$$

However, it is rather difficult to obtain accurate *DP_t_* values during the operation. In other words, it is impractical to predict the remaining life of a real transformer by using Equation (12). Therefore, an improved equation to predict the aging degree of polymer insulation of the transformer is required. An improved equation that can predict the *DP* value of transformer polymer insulation is proposed, which is shown in Equation (13).

(13) $$DP_{t}(t)=DP_{0}\cdot [1-\omega_{DP}(mc\%)\cdot (1-e^{-k_{DP}(mc\%)\cdot t})]$$

## 4. The Improved Aging Kinetics Model by Employing Frequency Dielectric Spectrum

On the condition that Equation (13) is desired to perform the analysis activities of the aging condition, a crucial basis is that the moisture content inside the polymer insulation materials is accurately determined. However, the determination of the moisture content inside an unknown cellulose sample is also difficult.

In view of the above issues, the existing researches reveal that dielectric frequency response might be used as an available approach to achieve the moisture analysis of polymer insulation materials [2,6,7,10,11]. Provided that the relevant parameter extracted from the analysis of FDS can be adopted to reflect the moisture content inside the cellulose insulation, the quantitative relationship between the extracted frequency dielectric response parameter and either *ω_DP_* (*mc%*) or *k_DP_* (*mc%*) can be established. Consequently, Equation (13) could be readily employed to carry out the aging condition prediction without the determination of the moisture content.

The purpose of this chapter is to find out the relevant frequency response feature parameter, which could characterize the moisture content but slightly affected by its aging condition. In the previous researches [14,15], the authors demonstrated that with the aggravating of the aging degree of transformer polymer insulation, the FDS curve changes remarkably in low-frequency regions, while remaining unchanged in high-frequency regions. Therefore, the frequency response information extracted from the high-frequency regions can be used to characterize the moisture content inside the transformer polymer insulation without being affected by its aging condition.

**Figure 4:**
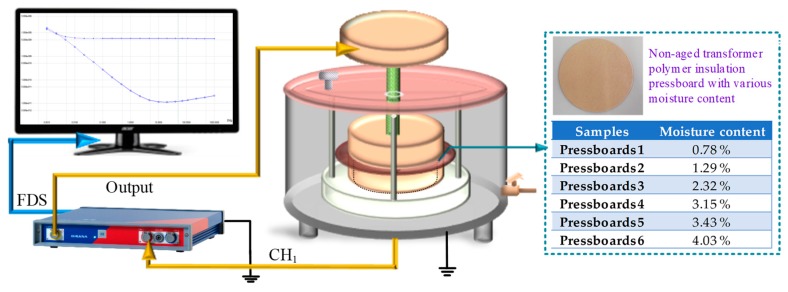
The frequency dielectric response test platform.

**Figure 5:**
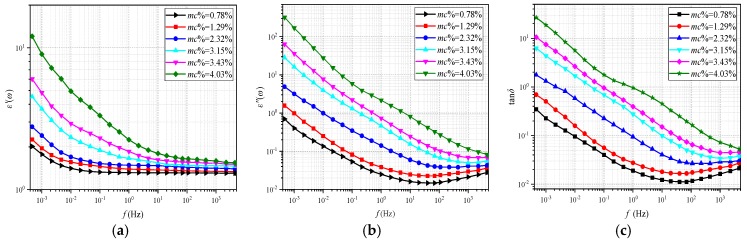
The ε*(*ω*) of non-aged polymer pressboards with various moisture contents. (**a**) *ε׳*(*ω*), (**b**) *ε׳׳*(*ω*), (**c**) tan*δ*.

In order to obtain the desired relevant parameters, the frequency dielectric response test is performed. It is launched in the three-electrode test cell under 45 °C. The test platform is shown in Figure 4. The FDS curves of non-aged cellulose pressboards with various *mc%* (including 0.78%, 1.29%, 2.32%, 3.15%, 3.43%, and 4.03%) are measured and plotted in Figure 5. Whereas dielectric loss (tan*δ*) is the ratio of imaginary part to the real part of ε*(*ω*), which is not affected by insulation geometry. Thus, the parameter extracted from the tan*δ* curve is more appropriate for various cellulose insulation materials.

As mentioned above, the tan*δ* curve in the high-frequency regions could merely reflect the information related to moisture content, and thus the selected high-frequency regions are defined as feature frequency regions, and a range of 100–1000 Hz is selected for this paper. In order to reduce errors in FDS data due to experimental operations, the integral operation (in 100–1000 Hz) is therefore utilized to extract the expected frequency response parameter. Equation (14) shows the calculation formula of these integral values (*IV*). The computed *IV* values are shown in Table 4.

(14) $$IV=\sum_{f_{a}}^{f_{b}}\tan\delta (f)\cdot f=\int_{100}^{1000}\tan\delta (f)df$$

**Figure 6:**
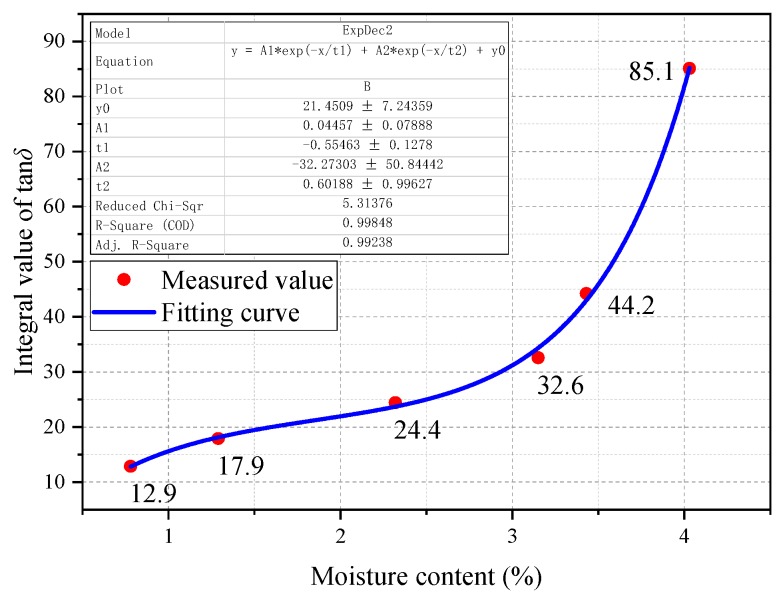
The fitting equation of *IV* vs. *mc%*.

An obvious exponential relationship of *IV* vs. *mc%* can be observed, and this viewpoint is proved by the fitting analysis. Its results are shown in Figure 6 and Table 5, respectively.

Form Table 5, the functional relationship between *mc%* and *IV* can be expressed by the means of *IV* = *f*(*mc%*), thus, the inverse function formula of *IV* could be expressed as *mc% = f* ^−1^(*IV*). Furthermore, from Table 3, provided that *ω_DP_* = *g*(*mc%*), and *k_DP_* = *p*(*mc%*), it means that the moisture content utilized in the formula of *ω_DP_* and *k_DP_* can be replaced by the inverse function of *IV*. Furthermore, if the *mc%* in the formula is offset and the quantitative relationship between *IV* and *ω_DP_*/*k_DP_* is established directly, the processed formula is written as *ω_DP_* = *g*[*f* ^−1^(*IV*)], and *k_DP_* = *p*[*f* ^−1^(*IV*)]. Then, the model for aging condition prediction based on *IV* can be derived, which is shown in Equation (15).

(15) $$DP_{t}(t)=DP_{0}\cdot [1-\omega_{DP}\cdot [f^{-1}\left(IV\right)]\cdot (1-e^{-k_{DP}\cdot [f^{-1}\left(IV\right)]\cdot t})]$$

**Figure 7:**
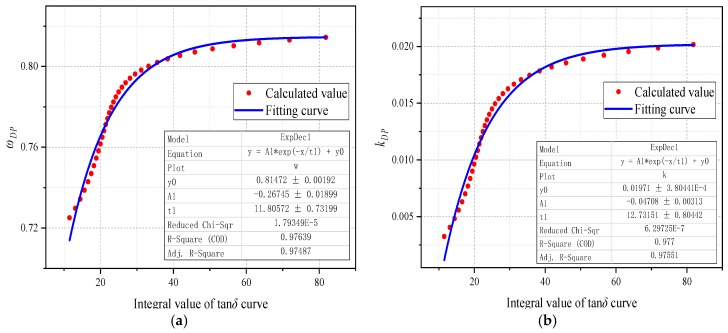
The fitting curves. (**a**) *ω_DP_* vs. *IV*, (**b**) *k_DP_* vs. *IV*.

The functional relationship between *IV* and *ω_DP_*/*k_DP_* is derived by simultaneous analysis of the equations shown in Table 3 and Table 5. Then, Figure 7 and Table 6 present the corresponding fitting curves and fitting equations, respectively. From Table 6, *ω_DP_* and *k_DP_* can be calculated by the integral value (*IV*) of the tan*δ* curve. The modified model introduced in Equation (15) will promote the practice of aging condition prediction of the unknown polymer samples on the condition that its moisture content is not determined.

## 5. A Modified Aging Kinetics Model for Aging Condition Prediction Including the Temperature Effect

In the previous chapter, the integral value *IV* was applied to calculate the value of *ω_DP_* and *k_DP_* corresponding to the same moisture content. Thus, the modified model considering the parameter *IV* can be applied to predict the aging condition of samples with various moisture contents. However, the values of *ω_DP_* and *k_DP_* are obtained by analyzing the variation law of *DP* vs. aging duration at 150 °C. Theoretically speaking, the proposed model is only suitable for transformer polymer insulation operating at 150 °C. This defect will greatly limit its versatility.

Fortunately, the time-temperature superposition theory [29,30] indicates that the effect generated by increasing temperature and prolonging the reaction duration on the molecular motion is equivalent. In other words, a similar degradation degree of the transformer polymer insulation under high temperature can be also observed in other conditions (both a lower temperature and longer duration). Such property can be expressed as follows, *t*_ref_·*T*_ref_ = *t*·*T*, *T*_ref_ (*T*) is regarded as the reference temperature (different operating or thermal aging temperature), *t*_ref_ (*t*) is the time (hours) required for the chemical reaction to reach a certain aging degree at the temperature of *T*_ref_ (*T*). Review the Arrhenius theory, which is utilized to interpret the variation law between chemical reaction rate and reaction temperature [17,18] as is shown in Equation (16).

(16) $$k_{i}=A\cdot e^{-\frac{E_{a}}{R\cdot T_{i}}}$$

where the *A* is the pre-exponential factor, which is related to the moisture content inside the polymer insulation material, *R* is a gas constant, *R* = 8.314 J/mol·K, *k*_i_ represents the chemical reaction rate of the same reaction under *i*-th types of temperature, and *E*_a_ is activation energy and assumed to be 103 kJ/mol [27]. In this paper, 423.15 K (150 °C) is regarded as the *T*_ref_. Therefore, the ratio of the reaction rates at the *T*_ref_ and *T* can be expressed by Equation (17), which is defined as shift factor *α*_T_.

(17) $$\alpha_{T}=\frac{T}{T_{ref}}=\frac{k}{k_{ref}}=e^{\frac{E_{a}}{R}(\frac{1}{T_{ref}}-\frac{1}{T})}$$

Thus, the formula for calculating the time *t* required to reach a given degree of reaction at temperature *T* can be represented by Equation (18) [25].

(18) $$t_{ref}=t\cdot \alpha_{T}=t\cdot e^{\frac{E_{a}}{R}(\frac{1}{T_{ref}}-\frac{1}{T})}$$

Obviously, relying on the simultaneous analysis of Equation (15) and Equation (18), the resulting modified model will be suitable for predicting the aging condition of transformer polymer insulation operating (or prepared) at different temperatures, which is presented in Equation (19).

(19) $$\left\{ \begin{array}{l} DP_{t}(t,IV,T)=DP_{0}\cdot [1-\omega_{DP}\left(IV\right)\cdot (1-e^{-k_{DP}\left(IV\right)\cdot t_{ref}})]\\ \omega_{DP}(IV)=-0.27\cdot e^{-IV/11.8}+0.81\\ k_{DP}(IV)=-0.047\cdot e^{-IV/12.7}+0.020\\ IV=\sum_{100}^{1000}\tan\delta (f)\cdot f=\int_{100}^{1000}\tan\delta (f)df\\ t_{ref}=t\cdot \alpha_{T}(T)\\ \alpha_{T}(T)=e^{\frac{E_{a}}{R}(\frac{1}{T_{ref}}-\frac{1}{T})} \end{array}\right.$$

**Figure 8:**
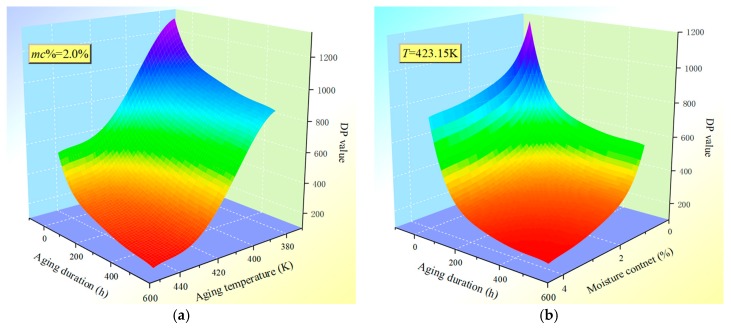
The *DP* prediction under different conditions. (**a**) *DP* vs. *t* and *T*, (**b**) *DP* vs. *t* and *mc*%.

It is obvious that the aging condition of the transformer polymer insulation in this model relies on three crucial factors (parameters), including aging duration *t* in the hour (at temperature *T*), initial moisture content (represented by integral value *IV*), and operating (thermal aging) temperature *T*. Moreover, the crucial basis of Equation (19) is that its FDS data (tan*δ*) is already known, and then, the prediction results of aging conditions at the reference temperature (150 °C) can be extended to different operating (thermal aging) temperatures by considering the shift factor shown in Equation (18). Thus, Equation (19) can be applied to predict the *DP* value of the transformer polymer insulation with different initial moisture content after *t* hours of operation (or thermal aging) at temperature *T*. The aging condition (*DP* value) of transformer polymer insulation at a different initial moisture (integral value) content and operating (thermal aging) temperature can be predicted by using Equation (19), respectively, and the resulting picture is presented in Figure 8. Figure 8a plots the *DP* vs. *t* and *T*, in the case of *mc*% = 2.0%. Figure 8b plots the *DP* vs. *t* and *mc*%, and *T* equal to 423.15 K.

## 6. Feasibility Investigation of the Proposed Modified Aging Kinetics Model

**Figure 9:**
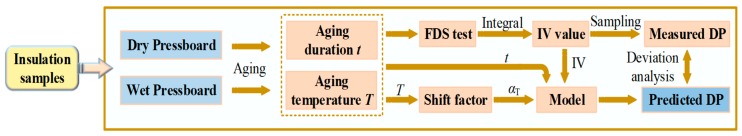
The prediction schedule of the *DP* of the transformer polymer insulation.

A novel modified aging kinetics model for predicting the aging condition (i.e., *DP* value) of transformer polymer insulation materials has been put forward. It is observed that once the FDS data, aging duration, and the aging temperature is determined, Equation (19) could be employed to predict the *DP* value immediately. In order to demonstrate the feasibility of the proposed model, the investigation experiments of *DP* prediction of transformer polymer insulation prepared in lab condition is performed. The prediction schedule is plotted in Figure 9.

In this chapter, several transformer polymer insulation pressboards with different insulation conditions are selected to verify the feasibility of the proposed model. The preparation schedule of these pressboards is referred to in Figure 1, and its details are described in Table 7. Pressboards 1–4 show low initial *mc%*, while pressboards 5–8 contain high initial *mc%*.

**Figure 10:**
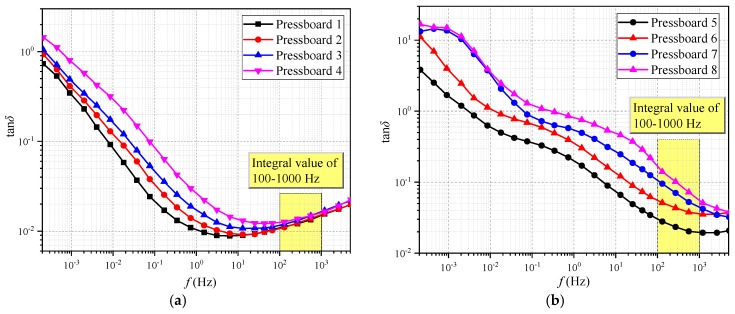
The Frequency Domain Spectroscopy (FDS) of polymer pressboards. (**a**) Dry pressboard, (**b**) wet pressboard.

Moreover, the FDS test is performed at 45 °C, the resulting tan*δ* curves corresponding to the above eight samples are presented in Figure 10. Afterwards, the integral values in a range of 100–1000 Hz are calculated based on these tan*δ* curves, and the calculated values are shown in Table 3 as well.

Substituting the parameter (*T*, *t*, and *IV*, shown in Table 7) into Equation (19), the relevant model parameters (including the *ω_DP_*, *k_DP_*, and *α*_T_) and predicted *DP* values of these pressboards with different initial moisture content, aging duration, and thermal aging temperature are obtained immediately and shown in Table 8.

**Figure 11:**
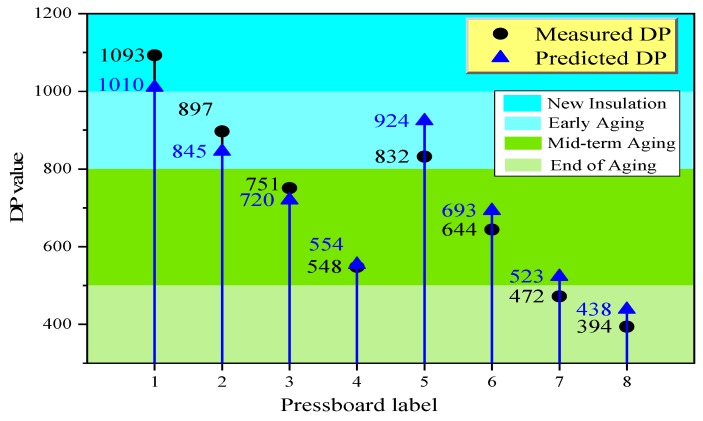
Comparison of the measured value and predicted value.

The formula of relative error is shown in Equation (20). It is found from Table 8 that the relative error between the predicted results and the measured results keep in 1%–12%, and the average value of these relative errors is equal to 7.41%. Furthermore, the estimation error in Table 8 is plotted in Figure 11, which is represented by the distance between the measured value and the predicted value. The comparison of the actual aging state and the predicted state of the prepared samples are also shown in Figure 11 according to the aging state division involving the operating experience.

(20) $$\mathrm{Relative}\;\mathrm{error}=\frac{\left|\mathrm{Predicted}\;\mathrm{value}-\mathrm{Measured}\;\mathrm{value}\right|}{\mathrm{Measured}\;\mathrm{value}}\times 100\%$$

It is worth noting that the reported fitting equations, models, and selected parameters significantly depend on the individual materials and material conditions used during the experiments (both for the oil and the pressboard). Fortunately, the parameters for describing the microstructure or physicochemical property of transformer polymer (cellulose) insulation materials follow similar variation rules (or laws) in the course of the degradation process. Therefore, if the proposed model is supported to perform the aging condition prediction of polymer insulation under other test conditions (such as the various thickness, density, etc., of experiment materials), the contained parameters should be revised in advance.

## 7. Conclusions

Since the moisture content inside the unknown sample is difficult to determine, the traditional aging kinetics model is thus not appropriate to perform the aging condition prediction of transformer polymer insulation materials. Fortunately, provided that the moisture content of the polymer materials could be characterized (replaced) by a certain feature parameter, the above issue will be solved naturally. According to this attempt, a modified aging kinetics model improved by employing the FDS technique is reported. The present analysis and contributions have led to the following conclusions.

1. The integral value of the tan*δ* curve (*IV*) can be utilized to characterize the moisture content inside the transformer polymer insulation materials accurately. Therefore, relying on the analysis of the relationship between the model parameters (*ω_DP_* and *k_DP_*) and *IV*, its functional relationship can be established;

2. Depending on the simultaneous analysis, this paper reported a modified aging kinetics model, which could be applied to perform the aging condition prediction of transformer polymer insulation materials under various test conditions, including aging duration, aging temperature, and initial moisture content. Moreover, the average relative error of prediction results of oil-immersed polymer (cellulose) pressboards is equal to 7.41%, which verified its accuracy and feasibility;

3. The reported fitting equations, models, and selected parameters significantly depend on the individual materials and material conditions used during the experiments. Therefore, if the proposed model is supported to be applied to other test conditions (such as the various thickness, density, etc., of experiment materials), the contained parameters should be revised in advance.

## Figures and Tables

**Table 1:** Primary parameters of both cellulose pressboard and oil used in the experiment.

Transformer Polymer Pressboard		Transformer Insulating Oil	
Brand	T_4_ Weidmann transformer pressboard	Brand	Karamay No.25 naphthenic mineral oil
Thickness	0.5 mm	Dielectric loss	4 × 10^−4^
Tensile strength	MD: 98 MPa, CMD: 47 MPa	Pour point	≤−45 °C
Density	0.96 g/cm^3^	Flash point	135 °C

**Table 2:** The details of the used parameters in the aging kinetics model.

*mc%*	0.78%		1.32%		2.07%		2.74%		3.82%	
	value	*EB* (±)	value	*EB* (±)	value	*EB*(±)	value	*EB* (±)	value	*EB* (±)
*ω_DP_*	0.73	2.36 × 10^−4^	0.75	1.38 × 10^−4^	0.78	4.45 × 10^−4^	0.79	6.13 × 10^−4^	0.81	6.99 × 10^−4^
*k_DP_*	0.0034	6.34 × 10^−4^	0.0074	2.76 × 10^−4^	0.012	1.25 × 10^−3^	0.016	2.36 × 10^−4^	0.019	1.41 × 10^−3^

**Table 3:** The fitting equation of *mc%* vs. *ω_DP_* and *mc%* vs. *k_DP_*.

Fitting Equation
$\omega_{DP}(mc\%)=-0.16\cdot e^{-mc\%/2.6}+0.85$
$k_{DP}(mc\%)=-0.033\cdot e^{-mc\%/3.3}+0.030$

**Table 4:** The parameters of integral value (*IV*) and *mc%*.

Parameters	Measured Value					
*mc%*	0.78%	1.29%	2.32%	3.15%	3.43%	4.03%
*IV*	12.9	17.9	24.4	32.6	44.2	85.1

**Table 5:** The fitting equation of *IV* vs. *mc%*.

Fitting Equation of *IV*
$\;IV(mc\%)=21.5+0.045e^{mc\%/0.56}-32.3e^{-mc\%/0.60}$

**Table 6:** The fitting equation of *ω_DP_* vs. *IV* and *k_DP_* vs. *IV*.

Fitting Equation
$\omega_{DP}(IV)=-0.27\cdot e^{-IV/11.8}+0.81$
$k_{DP}(IV)=-0.047\cdot e^{-IV/12.7}+0.020$

**Table 7:** The details of the prepared pressboards.

	Pressboard	Dry Pressboard				Wet Pressboard			
Parameters		1	2	3	4	5	6	7	8
Measured *mc*%		0.98%	1.02%	1.12%	1.15%	2.31%	2.93%	3.87%	4.12%
Measured *DP*_m_		1093	897	751	548	832	644	472	394
Aging temperature (*T*)/K		423.15	423.15	423.15	423.15	403.15	403.15	403.15	403.15
Aging duration (*t*)/h		24	72	96	168	96	168	264	336
Integral value (*IV*)		14.7	15.0	16.1	16.2	22.8	42.1	60.1	82.0

**Table 8:** Predicted results of the *DP* value of the prepared polymer pressboards.

	Pressboard	Dry Pressboard				Wet Pressboard			
Parameters		1	2	3	4	5	6	7	8
Parameters *ω_DP_*		0.738	0.740	0.747	0.747	0.776	0.807	0.813	0.815
Parameters *k_DP_*		0.0049	0.0052	0.0064	0.0065	0.012	0.018	0.019	0.020
Parameters *α*_T_		1.0	1.0	1.0	1.0	0.20	0.20	0.20	0.20
Measured *DP*_m_		1093	897	751	548	832	644	472	394
Predicted *DP*_p_		1010	845	720	554	924	693	523	438
Relative error		7.59%	5.80%	4.13%	1.09%	11.06%	7.61%	10.81%	11.17%
